# Caver Knowledge and Biosecurity Attitudes Towards White-Nose Syndrome and Implications for Global Spread

**DOI:** 10.1007/s10393-020-01510-y

**Published:** 2021-01-23

**Authors:** S. Salleh, K. Cox-Witton, Y. Salleh, Jasmin Hufschmid

**Affiliations:** 1 Department of Veterinary Biosciences, Faculty of Veterinary and Agricultural Sciences, Melbourne Veterinary School, The University of Melbourne, 250 Princes Highway, Werribee, VIC 3030 Australia; 2 Wildlife Health Australia, Suite E, 34 Suakin Drive, Mosman, NSW 2088 Australia; 3 The Childrens Hospital at Westmead, Cnr Hawkesbury Rd and Hainsworth St, Westmead, NSW 2145 Australia

**Keywords:** White-nose syndrome, Bats, Biosecurity, Caving community, Decontamination, *Pseudogymnoascus destructans*

## Abstract

White-nose syndrome (WNS), caused by the fungus *Pseudogymnoascus destructans*, has caused catastrophic declines of bat populations in North America. Risk assessment indicates that cavers could pose a risk for the spread of the fungus, however, information on cavers’ knowledge of WNS and their caving and biosecurity habits is lacking. An anonymous qualitative survey was completed by delegates (*n* = 134) from 23 countries at an international speleological conference in Sydney, Australia. Cavers indicated that they visit caves frequently (80.6% at least bimonthly), including outside of their own country, but 20.3% of respondents did not know about WNS prior to the conference. Some respondents were incorrect, or unsure, about whether they had visited caves in countries where *P. destructans* occurs (26.5%) or whether their own country was free of the fungus (7.8%). Although 65.9% of respondents were aware of current decontamination protocols, only 23.9% and 31.2% (when in Australian or overseas caves, respectively) fully adhered to them. Overall, cavers showed strong willingness to help prevent further spread of this disease, but further efforts at education and targeted biosecurity activities may be urgently needed to prevent the spread of *P. destructans* to Australia and to other unaffected regions of the world.

**Supplementary Information:**

The online version of this article (10.1007/s10393-020-01510-y) contains supplementary material, which is available to authorized users.

## Introduction

White-nose syndrome, caused by the fungus *Pseudogymnoascus destructans* (*Pd*), has caused a catastrophic decline of cave-dwelling bats in North America (Frick et al. 2010). Within two years of being detected at a single site, the fungus had spread over 200 km in all directions (Blehert et al. 2009). At the time of submission of this paper, 39 US states and seven Canadian provinces are affected (White-Nose Syndrome Response Team 2019). The fungus has also been detected in Europe and Asia but does not appear to be associated with the population-level effects seen in North America (Hoyt et al. 2016; Zukal et al. 2016).

It is thought that *Pd* was introduced to North America from Europe by anthropogenic means (Leopardi et al. 2015). WNS is a disease of hibernation, with affected bats rousing more frequently and succumbing to a combination of dehydration, starvation and electrolyte disturbances (Verant et al. 2014). Field studies have shown that *Pd* may survive for several years in cave environments, even in the absence of bats (Lorch et al. 2013), and in the laboratory, viable spores have survived for up to six years at low humidity (Hoyt et al. 2015). Spread of *Pd* between caves through contaminated objects is thus regarded as a significant risk (e.g. Shelley et al. 2013). North American guidelines (WNS Decontamination Team 2018) state that equipment used in affected regions should never be used in unaffected areas, irrespective of decontamination procedures. Boots, clothing and equipment used in a cave in an affected region should only be used in subsequent caves in other affected regions after appropriate decontamination procedures (Table 1). While bats are likely to be responsible for spread of the fungus over short distances, which may result in incremental spread across larger terrestrial areas (e.g. Maher et al. 2012), rapid spread over long distances is likely to be dependent on human involvement. A better understanding of the role of humans in the transmission of this disease is essential for the development and implementation of biosecurity strategies and to prevent further spread into unaffected regions.

**Table 1:** Decontamination Protocol for Equipment, Clothing and Shoes Used in Caving Activities, According to the WNS Decontamination Team Guidelines (WNS Decontamination Team 2018).

Procedure	Items
A: Scrub off any dirt upon entering and exiting a cave; seal equipment and clothing in a plastic bag or container to be cleaned and decontaminated offsite or at cave entrance	All caving equipment, shoes, clothes
B: Wash by hand or in a washing machine with conventional detergents	Submersible equipment, shoes, clothes
C: After using conventional detergents to help with removal of dirt, submerge gear in water of at least 50 °C (122 °F) for no less than 20 min	Submersible equipment, shoes, clothes
D: Treat with products such as alcohol (60% or greater); hydrogen peroxide (4.25%); sodium hypochlorite (8.25%); chlorhexidine gluconate (4.0%)^a^; or a quaternary disinfectant cleaner	Non-submersible equipment, shoes, clothes

^a^Included at time of survey, but removed from updated 2018 protocol due to variable testing results.

*Pseudogymnoascus destructans* is considered absent from Australia, based on surveillance (Holz et al. 2018) and a lack of observed clinical cases. The consequences of an incursion of *Pd* for native bats are uncertain, due to gaps in knowledge about hibernation ecology and the effect of local microclimatic conditions for cave-dwelling bat populations in Australia. However, Holz et al. (2019), and Turbill and Welbergen (2020) identified a significant potential risk to a number of Australian bat species, especially the critically endangered southern bent-winged bat (*Miniopterus orianae bassanii*). The former study identified several potential entry routes for *Pd*, including tourists, cave researchers and cavers. Cavers were considered the highest risk group, based on assumptions about frequency of visits to caves overseas, levels of knowledge and awareness of WNS and decontamination practices. However, the absence of objective data reduces the confidence in this judgement.

The greater caving community recognises the potential threat associated with *Pd* (e.g. White 2015)*.* However, there is no legal obligation in any country to decontaminate gear when moving between caving sites. The frequency of cavers entering Australia and the likelihood of visiting or returning cavers bringing potentially contaminated equipment into the country are currently unknown.

The aim of this work was to inform risk assessment and biosecurity measures to prevent the incursion of the exotic fungus *Pd* into Australia and between affected and unaffected regions in other parts of the world. The primary objective was to survey attendees at an international caving conference in Australia about their past and current practices relating to cave visitation, equipment decontamination and their understanding of the significance and transmission of WNS. A secondary objective was to investigate how willing delegates were to modify their biosecurity behaviour due to WNS.

## Methods

The study was a cross-sectional qualitative survey carried out in Sydney, Australia, during the 17th International Congress of Speleology between 23rd and 29th July 2017. All attendants of the congress, including Australian and overseas participants, were invited to participate using the participant invitation (see Supplementary Material 1). The questionnaire was designed by the authors assisted by external collaborators from the Australian Government Department of Agriculture and Water Resources and the Australian Speleological Federation.

To achieve the study objectives, data were collected on the following overarching questions:

1. What is the country of residence of respondents, the frequency of cave visits among delegates, both in Australia and overseas, and their reasons for visiting caves?
2. How much do cavers know about WNS?
3. What are the cleaning/decontamination habits of cavers for equipment and clothing?
4. What proportion of cavers indicated they would modify their biosecurity behaviours due to education about WNS provided before, or in association with, the conference?

The questionnaire contained 32 questions, 27 multiple-choice questions of which six had responses requiring open answers, three questions requiring numerical input and two questions being open-ended responses (Supplementary Material 2). The questionnaire was available to the participants on electronic devices using an online questionnaire (SurveyMonkey Inc., San Mateo, California) or paper forms located in an information booth, as well as a link to the survey website provided on a flyer included with the conference proceedings (Supplementary Material 1). The incentive to participate was entry into a prize draw to win a 1-year subscription to Australian Geographic magazine. Participants anonymously completed the questionnaire, with their identity kept separately from the questionnaire, and after entry to a prize draw, all personal information was destroyed. Each participant was invited to complete the survey only once. Before the conference, information on WNS was provided to attendees through the congress website, congress circulars and a letter sent to each delegate from the Australian Chief Veterinary Officer. A representative from Wildlife Health Australia gave an oral presentation on WNS during the conference (Cox-Witton et al. 2017).

The survey data were exported from the online survey software into a spreadsheet, where respondents answered with a numerical value range; the upper value was used for further analysis. To maintain anonymity, country or continent of origin is not specified in the results where an individual could be identified. A 2-proportions Fisher’s exact test was used to assess the difference in awareness of WNS and in error rate in judging *Pd*-status of visited countries between respondents from the countries where *Pd* is known to occur (henceforth referred to as *Pd*-positive countries) and not known to occur (*Pd*-negative countries). The relationships between Pd-status of country of origin and cleaning habits were explored by using likelihood-ratio chi-square tests. All statistical analyses were performed in Minitab 19 (Minitab® 19.2020.1). Statistical significance was set at *p* = 0.05.

Participants were included if they attended the conference and fully completed the survey. Not all questions were answered by every respondent, either because they chose not to respond or answered “not applicable”, or because dependent questions were not presented based on earlier answers. Survey responses were assessed based on the known status of *Pd* at the time of the conference (Table 2).

**Table 2:** List of Countries Known to Have *Pseudogymnoascus destructans* Based on the Scientific Literature at the Time of Publication.

Continent	Country	References
North America	USA	White-Nose Syndrome Response Team (2019)
	Canada	White-Nose Syndrome Response Team (2019)
Asia	China	Hoyt et al. (2016)
	Israel^b^	Hoyt et al. (2020)
	Japan^b^	Hoyt et al. (2020)
	Mongolia^b^	Hoyt et al. (2020)
Europe	Austria	Burger et al. (2013)
	Belgium	Puechmaille et al. (2011)
	Czech Republic	Martínková et al. (2010)
	Denmark	Puechmaille et al. (2011)^a^
	Estonia	Puechmaille et al. (2011)
	France	Puechmaille et al. (2010)
	Georgia^b^	Hoyt et al. (2020)
	Germany	Wibbelt et al. (2010)
	Hungary	Wibbelt et al. (2010)
	Italy^b^	Garzoli et al. (2019)
	Latvia	Zukal et al. (2016)
	Luxembourg	Mestdagh et al. (2012)
	Netherlands	Puechmaille et al. (2011)
	Poland	Puechmaille et al. (2011)
	Portugal	das Neves Paiva-Cardoso et al. (2014)
	Romania	Puechmaille et al. (2011)^a^
	Russia	Zukal et al. (2016)
	Slovakia	Martínková et al. (2010)
	Slovenia	Zukal et al. (2016)
	Switzerland	Wibbelt et al. (2010)
	Turkey	Puechmaille et al. (2011)^a^
	Ukraine	Puechmaille et al. (2011)
	United Kingdom	Barlow et al. (2015)
Antarctica		Santiago et al. (2015)

^a^Photographic evidence only

^b^Indicates records that were not known at the time of a survey at the international caving congress Speleo 2017, Sydney, Australia.

## Results

**Figure 1:**
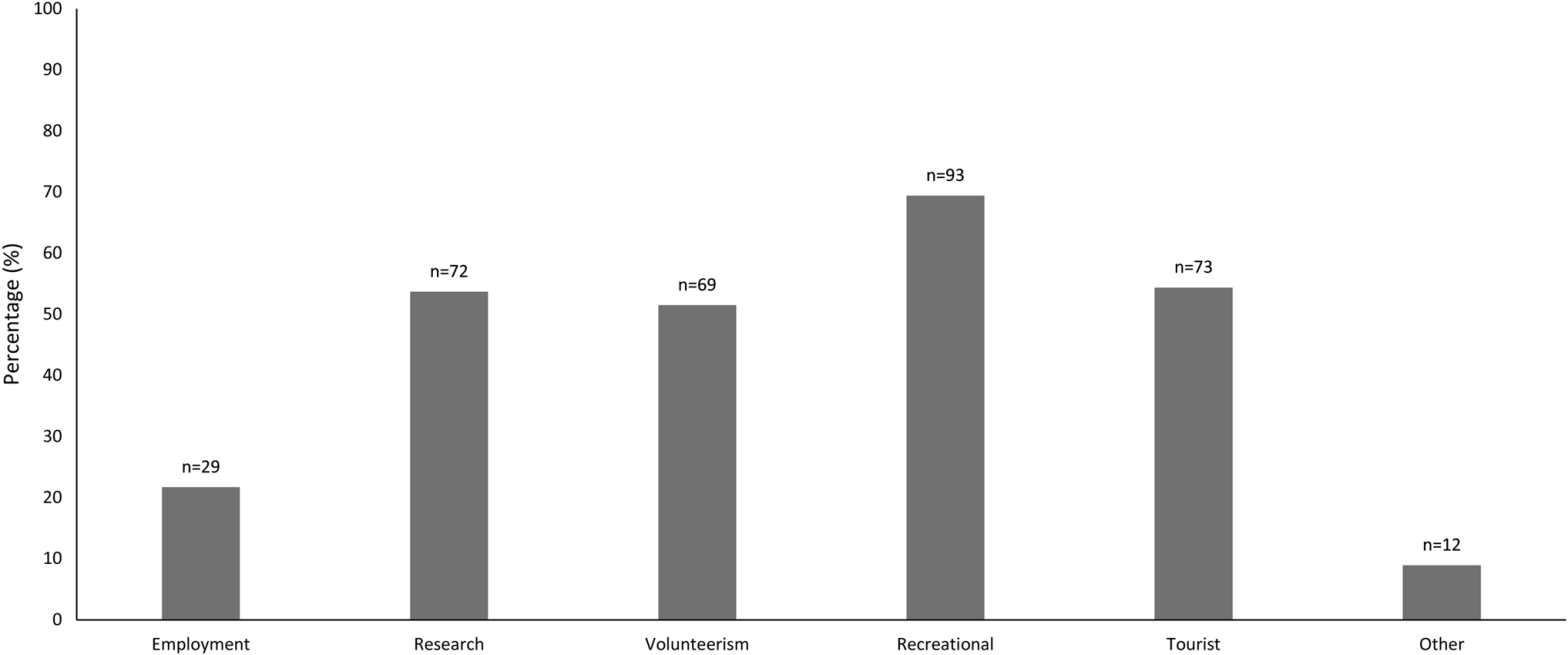
Reasons for caving among respondents (*n* = 134) to a survey at the international caving congress Speleo 2017. *n* = sample size.

A total of 462 people from 47 different countries attended the congress, and 137 participated in the study, giving a response rate of 29.7%. Three surveys were excluded because the respondent only answered the first few questions, resulting in a total of 134 surveys for analysis. Twenty-three countries of origin were represented in the survey. Australians made up the largest participant group at almost 40%, followed by North America, Europe, Asia, South America and Africa, in that order (see Table 3 for details). Recreation was the most common reason for caving, followed by tourism, research, volunteerism and employment (Fig. 1). Other reasons included cave rescue, cave ownership, and surveying, mapping or exploration.

**Table 3:** Country of Residence of Respondents to a Survey Conducted Among Delegates of Speleo 2017 Congress in Sydney, Australia.

Continent	Percentage of respondents (%)	Country	Number of participants
Africa	0.7	South Africa	1
South America	1.5	Brazil	2
North America	29.8	USA	37
		Canada	2
		Mexico	1
Asia	6.0	Indonesia	5
		Philippines	3
Australia	39.6	Australia	53
Europe	22.4	Germany	5
		Italy	5
		Croatia	2
		Slovenia	2
		Spain	2
		The Netherlands	2
		Belgium	1
		Czech Republic	1
		France	1
		Hungary	1
		Luxembourg	1
		Norway	1
		Portugal	1
		Russia	1
		Serbia	1
		Slovakia	1
		Sweden	1
		UK	1
Total	100		134

**Figure 2:**
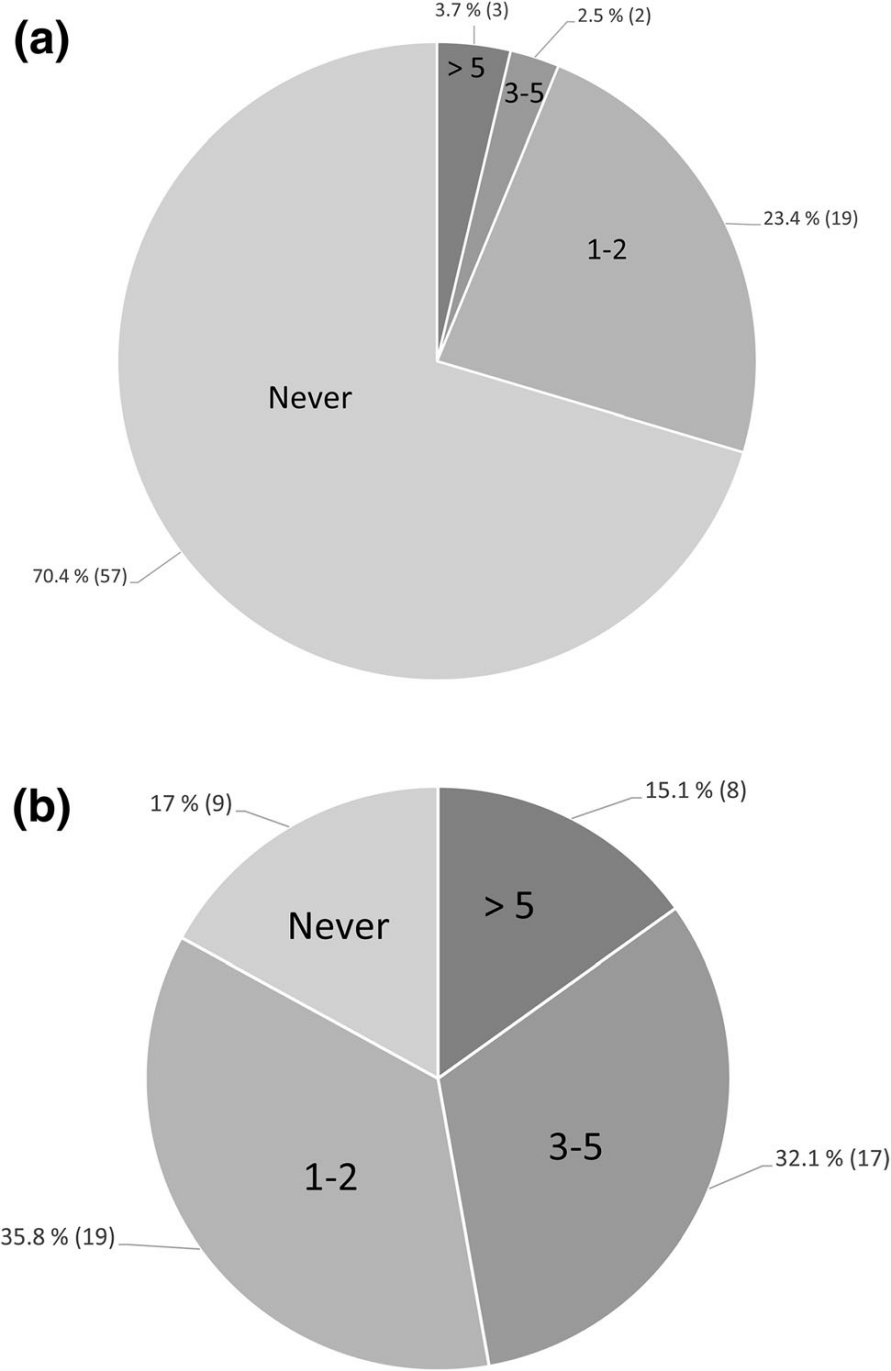
Number of times overseas visitors (*n* = 81) had visited Australian caves previous to current visit (**a**) and Australian respondents (*n* = 53) had caved overseas (**b**), based on a survey at the international caving congress Speleo 2017. *n* = sample size. Numbers in brackets indicate number of respondents.

Most respondents (80.6%, 108/134) visit caves in their own country weekly to every 2 months, with the remainder visiting once or twice a year (18.6%, 25/134) or not at all (0.75%, 1/134). In 2016, participants had visited a median of 11 caves (mean = 17.8; range: 0–100) in their own countries, and 46.2% (62/134) had visited caves in countries other than their own, with a median of five caves (range 1–100). Just under a third of overseas visitors had previously visited caves in Australia (Fig. 2a) and most Australians had visited caves overseas at some point in the past (Fig. 2b). Almost three-quarters of all respondents (71.5%, 98/137) had previously visited caves in a country that is now known to have *Pd.*

### Awareness and Knowledge of WNS

Although the vast majority (96.3%, 129/134) of participants reported that they knew about WNS at the time of the survey, only 79.7% (106/133) knew about the disease prior to the conference. Of those who had not heard of WNS before the congress, 18.5% (5/27) were from *Pd-*positive countries, including two North Americans. A substantial number of these (44.4%, 12/27), including seven Australians, had previously visited caves in *Pd*-positive countries. Almost half (48.9%, 63/129) of those with some knowledge of WNS answered that they know “a lot” or are “experts” in this disease, while 9.3% (12/129) “did not know much”. All those self-identifying as “experts” came from the USA, whereas those who felt they knew “not much” came from Australia (7/129) or elsewhere (5/129).

Of those who said they had heard of WNS, 84.4% (108/128) reported that they knew the status of WNS in their country. However, 4.7% (6/128) of these respondents incorrectly identified their country as free of *Pd*, even though four of these had identified as “knowing a lot” about WNS. A further 3.1% (4/128) from *Pd*-positive countries (all European) were unsure of their country’s status. Among Australian respondents, 17.6% (9/51) were not sure whether WNS or *Pd* occurred in Australia. Most respondents (76.6%, 98/128) were certain about whether they had visited a *Pd*-positive country. However, 13 respondents who said they were sure they had never visited caves in countries with Pd (34.2%, 13/38) had in fact done so based on their other answers. In two cases, the country visited was Canada, the other countries were the USA, Europe or China; one respondent had simply reported to have visited caves “all over the world”. In addition, 70% (21/30) of respondents who did not know whether they had visited caves in a *Pd*-positive country, had done so. Respondents from *Pd*-positive countries were better at judging whether they had visited other countries with *Pd* than those from *Pd*-negative countries (17.1% difference in error rate, 95% CI = 30.7, 3.6%; *p* = 0.024).

### Use and Decontamination of Caving Equipment in Caves Outside of Australia

Just over a third of respondents (34.1%, 44/129) were unfamiliar with current decontamination protocols. Five of those were resident in countries where *Pd* is known to occur. In addition, 5.5% of respondents (7/128) answered that *Pd* could not be spread easily between caves on equipment (boots, clothing and other equipment), all but one of these being from the USA. Similarly, 14.1% (18/128) were unsure whether WNS could be spread easily on such equipment, including 13 delegates from countries where *Pd* is known to occur. The vast majority of respondents, 96.9% (123/130), use their own caving equipment when visiting caves in their own country. Similarly, 80.2% (97/121) of respondents take their own gear when visiting caves in other countries.

Outside of Australia, 9.1% (10/110) of respondents dispose their equipment immediately after use in a cave, and 21% (21/100) of the remainder have different sets of equipment for different caves. Most respondents, 94.9% (74/78), who reuse equipment between different caves have some form of decontamination routine. The majority (61.5%, 48/78) stated that they always clean their equipment, while 21.8% (17/78) do so occasionally (every 2–3 trips), 11.5% (9/78) do so rarely (less than every 3 trips) and 5% (4/78) never clean their equipment. The *Pd*-status of the country of residence of respondents had no significant effect on these responses (likelihood-ratio chi-square = 5.42; df = 3; *p* = 0.143), but all of the respondents who stated that they never clean their equipment came from *Pd*-negative countries (see also Table 4).

**Table 4:** Frequency of Cleaning/Disinfecting of Caving Equipment and Clothing by Respondents Who Reuse Equipment Between Caves, Based on Whether They Come from Countries With or Without (*Pd*) (*Pseudogymnoascus destructans*), and Geographic Location of Caves.

Frequency of cleaning	Caves outside of Australia			Caves in Australia^a^		
	Percentage of respondents (%) (number)			Percentage of respondents (%) (number)		
	Pd present	Pd absent	Total	Pd present	Pd absent	Total
Always	67.7 (23)	56.8 (25)	48	61.1 (11)	67.9 (36)	47
Occasionally	23.5 (8)	20.5 (9)	17	22.2 (4)	18.9 (10)	14
Rarely	8.8 (3)	13.6 (6)	9	5.6 (1)	5.7 (3)	4
Never	0 (0)	9.1 (4)	4	11.1 (2)	7.5 (4)	5
Total	100 (34)	100 (44)	78	100 (18)	100 (53)	71

^a^Includes respondents who have caved in both Australia and overseas.

Only a small percentage of respondents, 11.5% (9/78), are fully adherent to the published guidelines for decontamination of caving equipment, using all four methods (see Table 1). Another 14.1% (11/78) use three of the recommended methods, 43.6% (34/78) use two and 23.1% (18/78) use only one method; 7.7% (6/78) do not use any of the methods. By far the most common combination of methods is physical removal and use of conventional cleaners (44.4% of all approaches). Respondents from *Pd*-positive countries were more likely to use a greater number of decontamination methods than those from *Pd*-negative countries (likelihood-ratio chi-square = 19.22, df = 4; *p* = 0.001) (Table 5). All of those using the full decontamination protocol were from the USA. Of those respondents not using any of the stipulated methods, only one was from a *Pd*-positive country; they used methods not stipulated in the protocol (“dishwasher and washing machine”).

**Table 5:** Number of Methods Used for Cleaning/Disinfecting of Caving Equipment and Clothing by Respondents Who Reuse Equipment Between Caves, Based on Geographic Location of Caves, and Whether They Come from Countries With or Without (*Pd*) (*Pseudogymnoascus destructans*).

Number of methods used	Caves outside of Australia			Caves in Australia		
	Percentage of respondents (%) (number)			Percentage of respondents (%) (number)		
	Pd present	Pd absent	Total	Pd present	Pd absent	Total
4	26.5 (9)	0 (0)	9	22.2 (4)	0 (0)	4
3	17.6 (6)	11.4 (5)	11	33.3 (6)	5.8 (3)	9
2	35.3 (12)	50 (22)	34	27.8 (5)	59.6 (31)	36
1	17.6 (6)	27.3 (12)	18	5.6 (1)	26.9 (14)	15
0	2.9 (1)	11.4 (5)	6	11.1 (2)	7.7 (4)	6
Total	100 (34)	100 (44)	78	100 (18)	100 (52)	70

### Equipment Use and Cleaning in Australian Caves

Looking specifically at cave visits in Australia, a total of 89 respondents indicated that they had caved in Australia, including 38 overseas visitors. This included 20 overseas visitors who had not caved in Australia before the current visit. The trends for cleaning of equipment were broadly similar for caves in Australia to that described above for overseas caves (see Tables 4 and 5 for detail and breakdown by *Pd*-status), but there were some notable differences between Australians and overseas visitors. None of the Australians dispose of their gear after visiting a cave, compared with 15.8% (6/38) of overseas visitors (95% CI for difference: 4, 27%; *p* = 0.005). Fewer Australian than overseas respondents change equipment between caves (9.8% vs 21.9%, respectively), but this was not statistically significant (95% CI for difference: − 4.4, 28.5%; *p* = 0.199).

There was also some evidence for different behaviour depending on whether caving occurred in Australian or overseas caves. All of the cavers from *Pd*-positive countries clean their equipment between caves when outside of Australia, but two do not when in Australia. Likewise, seven respondents (four Australians and three overseas visitors) only dispose of equipment when visiting caves outside of Australia and five respondents (two Australians and three Americans) only use different sets of equipment when caving outside of Australia. On the other hand, five overseas visitors dispose their equipment after frequenting a cave in Australia, but not when caving elsewhere.

Free comment responses to questions about decontamination habits are provided in Supplementary Material 3.

### Change in Behaviour

Of those respondents who had heard of WNS before the congress, 65% (69/106) reported to have changed their behaviour in relation to visiting caves (choice of caves visited, use of gear, equipment cleaning/disinfecting protocol) as a result of finding out about the disease. Approximately half of the respondents (50.0%, 67/134) answered that they planned to modify their future behaviour relating to cave visits because of information about WNS they received at the congress. The main reason given was a desire to protect bats in their own or other countries. Some did not see any need for change because they were not likely to visit caves in affected areas or because bats in their country were “resistant to WNS” (Europe) (15.6%, 10/64). Several of those giving the latter answer did, however, specify that they would change their behaviour if they visited caves where there was a risk that they could spread WNS to unaffected areas. One person reported an inability to (financially) afford modification of behaviour.

## Discussion

The objective of the present study was to provide information about caving habits, knowledge about *Pd* and WNS among the caving community, and current biosecurity habits associated with visiting caves in Australia and overseas. It is possible that those attending an international caving congress are among the more engaged members of the caving community, however, in that case, any bias in relation to the survey would most likely result in an overestimate of awareness about WNS and biosecurity measures. It is likely that a congress may predominantly attract cavers from countries with higher economic status, but cavers from wealthier countries are perhaps also more likely to travel for caving, and thus more likely to transfer *Pd* between regions.

Approximately half of the countries represented at the congress were represented in the survey. Unsurprisingly, given the location of the congress, the country with the highest number of delegates responding to the survey was Australia, however, substantial numbers were obtained from other geographic regions, especially North America and Europe. More respondents from Asia would have been useful, especially China where *P. destructans* has recently been identified. Nevertheless, we believe that the survey respondents constitute a reasonable representation of the congress population, allowing for some useful conclusions to be drawn.

Most respondents indicated that they visited caves frequently, and many had visited caves in a country that is now known to have *Pd*. However, not all respondents were aware of WNS. Unsurprisingly, perhaps, those with no knowledge of WNS before the congress were almost all from countries where WNS is not present (e.g. Australia) or where *Pd* has little population-level impact (e.g. Europe) (Wibbelt et al. 2010). However, given the extent of the WNS epidemic and associated publicity and education in North America, it is concerning that two North American delegates reported that they had not heard of WNS before registering for the congress. Although most North Americans showed a good understanding of the disease and associated risks, this result suggests that education messages have not reached some of the targeted stakeholders in the most acutely affected region.

One of the key findings was significant uncertainty among the caving community about the *Pd*-status of different countries. In addition, some overestimated their WNS awareness. Accurate, easily accessible information on *Pd*-status is critical to allowing cavers to mitigate their risk of *Pd* spread. Information on the global spread of *Pd* is not easy to access as it generally requires a review of individual reports in the scientific literature. To the authors’ best knowledge, there is currently no central, easily accessible international information site on the *Pd*-status of countries and regions worldwide.

Most of those declaring that they were unaware of appropriate decontamination protocols were resident in *Pd*-negative countries. It is likely that caving communities in geographic areas where *Pd* is not known to occur have less exposure to discussions about WNS and published guidelines on decontamination. The remaining respondents may also not have seen any need to decontaminate their equipment in the absence of the fungus, however, there is a potential risk if they travel into affected regions and return with equipment that has not been properly cleaned. Somewhat worryingly, six respondents from the USA believed that *Pd* could not be easily spread between caves on equipment. There is, however, broad agreement by researchers (e.g. Ballmann et al. 2017, Wibbelt 2018) and wildlife managers (Sleeman 2011) on the potential of such spread.

Most respondents declared that they use their own equipment within and outside their country of residence. This included 70.3% of the US cavers. It is worth noting that the updated North American guidelines recommend that no caving equipment should be used between the USA and other countries (WNS Decontamination Team 2018); however, this was not specified in the 2011 guidelines so the results could have been different if the survey had been conducted after 2018. Nevertheless, coming from a *Pd*-positive country did appear to result in more informed biosecurity habits, perhaps as a result of greater exposure to educational materials on WNS decontamination practices. The only respondents to never clean their equipment when caving outside of Australia were from *Pd*-negative countries. Somewhat worryingly though, all those cavers had caved in *Pd*-positive countries and tended to use their own equipment when travelling to caves outside of their own country.

Only a minority of respondents (all from North America) follow the full current decontamination protocol, although cavers from *Pd*-positive countries were more likely to use more decontamination steps than those from *Pd*-negative countries. Current guidelines suggest that the two most commonly used decontamination steps, physical removal of dirt and conventional cleaner, alone are not effective in fully eliminating *P. destructans* (Shelley et al. 2013). Based on the relatively small number of open-ended comments by respondents, there may be a lack of awareness of the effectiveness of different cleaning methods, but there may also be concern about the effect of disinfectants on caving equipment and the effort involved in conducting the full decontamination protocol. Only a relatively small number of respondents dispose their equipment after visiting a cave or have different sets for different caves. This is not surprising, given the cost of caving equipment, and it seems likely that those who dispose their equipment use hired or borrowed equipment. Considerations around risk of *Pd*-contamination, on the other hand, may be the reason that some respondents used different sets of gear between caves outside of Australia, but not in Australia, which is considered to be *Pd*-negative.

Overall, respondents indicated that they already had, or would be, changing their caving behaviour as a result of learning about WNS. Nevertheless, a third of Europeans felt no need to change their behaviour because bats in their countries were “resistant” (sic) to WNS (although some said they would change behaviour if visiting other areas). There may be a degree of complacency among European cavers due to the lack of significant impact of WNS on bats in their country.

For Australia, the present survey suggests that there is indeed a risk of introduction of *P. destructans* into the country through the caving community, from both Australian and overseas cavers. While there are still unknowns regarding the consequences of introduction of *Pd* to Australia, there is significant concern that WNS could have detrimental impacts on hibernating, cave-dwelling bat species, including the critically endangered southern bent-wing bat (Holz et al. 2019; Turbill and Welbergen 2020). Mitigation of this risk could be achieved through continuing widespread education of the caving community through organisations such as the Australian and European Speleological Federations, as well as raising public awareness more generally through the media. Specific to Australia, on arrival to the country, there could be clear and visible messaging about the risk to bat conservation from having visited overseas caves, along with clear recommendations on biosecurity protocols. There are currently no specific questions about caving equipment at entry points to Australia, however, as part of broader biosecurity, arrivals are asked to declare on their Incoming Passenger Card any items that may be contaminated with soil and presence in wilderness areas in the last 30 days (Australian Border Force 2020). Given the confusion among cavers about their countries’ *Pd*-status, centralised, easily accessible information such as a regularly updated website would be very useful.

This survey focused on risks associated with the caving community; however, there may be additional risks for anthropogenic spread of *P. destructans* associated with other cave visitors and tourists. While outside the scope of this study, investigation of these other significant potential risk groups would be worthwhile.

## Conclusion

Based on current awareness of WNS and associated biosecurity requirements among the domestic and international caving community, there is, despite extensive efforts by speleological societies to educate cavers, still a significant risk that *Pd* will be introduced to Australia. Further, and possibly more widespread, education of the caving community and the general public, as well as more restrictive border controls, are likely to reduce this risk.

## Supplementary Information

Below is the link to the electronic supplementary material.

Supplementary material 1 (DOCX 604 kb)
