# Supplementary material for: Caver Knowledge and Biosecurity Attitudes Towards White-Nose Syndrome and Implications for Global Spread
Source: Ecohealth. 2021 Jan 23;17(4):487–97. doi: 10.1007/s10393-020-01510-y (PMC8192400; doi:10.1007/s10393-020-01510-y)
Supplement: Supplementary file 1 — Supplementary material 1 (DOCX 604 kb) [file 10393_2020_1510_MOESM1_ESM.docx]

**Supplementary Materials 1:** Flyer distributed to participants of Speleo 2017, an international caving congress in Sydney, Australia.

**Supplementary Materials 2.** Survey questions (and possible answers) asked of delegates to Speleo 2017 Congress in Sydney in 2017 about their knowledge of white nose syndrome (WNS) and biosecurity habits.

| **Survey question** | **Possible answers** |
| --- | --- |
| 1. Where do you live? (multiple choice) | Australia; USA; Canada; Europe; Asia; Other (specify) |
| 1. How often have you visited caves overseas (Australian only) | once or twice (rarely); three to five times (occasionally); more than five times (frequently); never |
| 3) Which country do you live in? (open ended) | open-ended (for Q1 Europe and Asia) |
| 4) How often have you visited caves in Australia prior to this visit? | once or twice (rarely); three to five times (occasionally); more than five times (frequently); never |
| 5) How many caves did you visit in Australia in the year 2016? | open-ended (numerical value) |
| 6) How often do you visit caves in your own country? | once or twice a year (rarely); month to every two months (occasionally); weekly (frequently); never |
| 7) In the year 2016, how many caves did you visit in your own country? | open-ended (numerical value) |
| 8) Which other countries have you visited caves in? | open-ended response |
| 9) In the year 2016, how many caves did you visit in countries other than your own? | open-ended response (numerical value) |
| 10) What is your purpose for visiting caves (multiple choice) | part of employment; conduct research; volunteer work; recreational caving; tourist; other (specify) |
| 11) Do you take your own gear with you when you visit caves in your own country (this includes clothes, footwear and all other equipment taken to cave locations) | yes; no; not applicable |
| 12) Do you usually take your own gear with you when you visit caves in countries or regions other than your own? | yes; no; not applicable |
| 13) When visiting caves in Australia in the past (excluding conference-associated field trips), did you throw away your gear immediately after use in caves? | yes; no; not applicable |
| 14) Did you have separate sets of gear for different caves | yes; no |
| 15) How frequently do you clean/disinfect your gear after leaving a cave? | always; occasionally, every 2-3 trips; rarely, less than every three trips; never |
| 16) Caves in Australia: how do you clean/disinfect your gear (including ropes, clothing, shoes, harness etc) (Multiple choice – tick all that appropriate) | physical removal of dirt/sediment; use of conventional cleaners (soap, laundry liquid); submersible equipment: in hot water at 55 °C/131 °F for at least 20 minutes; non-submersible equipment: disinfectant containing either chlorine/chlorhexidine, hydrogen peroxide, >60% alcohol or a quaternary disinfectant (e.g. Lysol); never clean/disinfect my gear; other (specify) |
| 17) When visiting caves outside Australia, did you throw away your gear immediately after use in caves? | yes; no; not applicable |
| 18) Did you have separate sets of gear for different caves? | yes; no |
| 19) How frequently do you clean/disinfect your gear after leaving a cave? | always; occasionally, every 2-3 trips; rarely, less than every three trips; never |
| 20) Caves outside Australia: How do you clean/disinfect your gear (including ropes, clothing, shoes, harness et) (Multiple choice – tick all that appropriate) | physical removal of dirt/sediment; use of conventional cleaners (soap, laundry liquid); submersible equipment: in hot water at 55 °C/131 °F for at least 20 minutes; non-submersible equipment: disinfectant containing either chlorine/chlorhexidine, hydrogen peroxide, >60% alcohol or a quaternary disinfectant (e.g. Lysol); never clean/disinfect my gear; other (specify) |
| 21) Have you changed the way you deal with your caving equipment, including decontamination, for the first time in association with this conference and associated field trips? | yes; no |
| 22) Have you heard of White Nose Syndrome (WNS) in bats? | yes; no |
| 23) Did you first hear about WNS in conjunction with the Speleo 2017 congress? | yes; no |
| 24) How much do you feel you know about WNS? | not much; a little; a lot; expert |
| 25) Are you familiar with the current recommended decontamination protocols for WNS (e.g. US National Decontamination Protocol; or UK Guidance for Bat Workers and Cavers) | yes; no |
| 26) Is WNS a risk to bats in Australia? | yes; no; I don’t know |
| 27) WNS (and/or the fungus causing WNS) is present in my country of residence | yes; no; I don’t know |
| 28) I have previously visited caves in countries that are now known to have WNS (and/or the fungus causing WNS) | yes (specify country); no; I don’t know |
| 29) WNS is a significant danger to the bat populations in my country of residence | yes; no; I don’t know |
| 30) WNS can easily be spread between caves on boots, clothing and other caving equipment | yes; no; I don’t know |
| 31) I modified my behaviour related to visiting caves (e.g. choice of caves visited, use of gear, decontamination procedures) since hearing about WNS through means other than the 2017 Speleo Congress | yes; no |
| 32) Are you likely to change your future behaviour related to visiting caves because of the WNS information provided to you through the Speleo 2017 congress? | yes (why?); no (why?) |

**Suppplementary Materials 3.** Additional (free comment) survey answers regarding decontamination protocols of equipment and clothing by delegates to Speleo 2017. Each response was given once.

**Caving in Australia:**

- “Hose down or hand-wash cordura and rope, cotton cloth and tap (sic)”
- “Tried metho once but destroyed my gear”
- “Have not used disinfectant unless required”
- “I wash my cave guide uniform frequently – no caving gear”
- “Submersible gear soaked in quaternary disinfectant”

**Caving outside of Australia:**

- “microwave”
- “wash clothes, but never helmet, only wash harness if gets really muddy”
- “do not use disinfectant unless required”
- “visiting show caves only, no gear” (this respondent had indicated one of the suggested cleaning methods)
- “I almost always cave in the North American WNS-everywhere zone, where moving WNS to caves where it is not already present is essentially impossible”
- “water”
- “Pressure washer and washing machine sometimes”
- “Hosing down”
- “Dishwasher and washing machine” (this respondent had indicated none of the suggested methods)
- “machine wash at low temperature”
- “in water at 40 C for 40 minutes”
- “submersible equipment soaked in quaternary disinfectant” (n=1)
